# Linking cell biology and ecology to understand coral symbiosis evolution

**DOI:** 10.1371/journal.pbio.3002593

**Published:** 2024-04-11

**Authors:** Niels J. Dingemanse, Annika Guse

**Affiliations:** Faculty of Biology, Ludwig-Maximilians-Universität Munich, Munich, Germany; University of Oxford, UNITED KINGDOM

## Abstract

Understanding the evolution of coral endosymbiosis requires a predictive framework that integrates life-history theory and ecology with cell biology. This Perspective article argues that the time has come to bridge disciplines, and use a model systems approach to achieve this aim.

## Teaser

Understanding the evolution of coral endosymbiosis requires a predictive framework that integrates life-history theory and ecology with cell biology. The time has come to bridge disciplines and use a model systems approach to achieve this aim.

Symbiosis between corals and photosynthetic dinoflagellates involves nutrient exchanges that enable both partners to prosper in nutrient-poor marine environments. Dinoflagellates transfer organic nutrients (e.g., sugars, lipids, amino acids) to their hosts and receive inorganic nutrients (e.g., ammonium, nitrate, phosphate) in return. This bidirectional transfer is the primary driver of coral reef productivity and biodiversity.

Corals and dinoflagellates form an endosymbiosis in which dinoflagellates reside inside the coral’s endodermal cells. Symbiosis is reestablished in each generation as coral larvae engulf symbionts from the environment and integrate them into host cell functions. Forming a stable partnership requires multiple intertwined biological processes, including phagocytosis, modulation of host cell immunity, vesicular transport, metabolism, and nutrient exchange [1]. Uncovering the cellular mechanisms that determine this partnership is key to understanding coral ecology and evolution.

In nature, the dinoflagellate symbionts that associate with corals are highly diverse, even within coral colonies and species [2]. However, host–symbiont pairings are nonrandom and can be stable over the lifetime of individual organisms and over generations because ecological conditions affect which host–dinoflagellate combinations are optimal. Accordingly, symbiont-free larvae and juvenile coral polyps are selective, picking partners that are most suited for coping with their specific environment [1]. Likewise, corals recovering from bleaching (loss of symbionts) can engage with different symbionts than those that were present before the bleaching event.

But what are optimal symbiont–host pairings? Studies focusing on the molecular mechanisms underpinning host–symbiont interactions provide clues as to why some pairs may be “better” than others [1]. For example, nutrient transfer efficiency and the composition of exchanged nutrients differ between host–symbiont combinations. Some symbiont strains fix and transfer more carbon to their host and obtain more nitrogen in return. Indeed, host-induced nitrogen limitation likely regulates symbiont growth during symbiosis, and because hosts differ in what they provide, it is not surprising that symbiont performance depends on host partner type. Moreover, corals are sterol auxotrophs and depend on their symbionts to satisfy their (chole)sterol needs. Furthermore, the corals’ sterol profile varies depending on the associated symbiont strain, and as sterols are essential building blocks for cell membranes, differences in sterol transfer might affect coral growth and reproduction [1]. However, we have yet to understand the exact molecular interactions involved and what defines optimal versus suboptimal pairings. Because we do not know how cellular interactions and metabolic exchanges vary, we cannot fully understand how coral symbioses function across ecological space.

**Fig 1:**
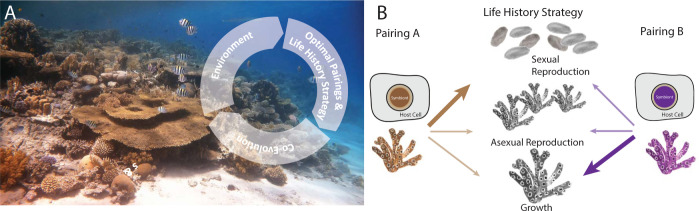
Cell biology underpins the ecology and evolution of coral symbiosis. (**A**) An eco-evolutionary feedback loop, whereby environmental conditions determine the optimal symbiont–host pairing and life-history strategy to maximize fitness of both, which leads to coevolution of partners shaping coral reef ecosystems (Photo credit: Malik Naumann). (**B**) Distinct life-history traits employed by corals are likely underpinned by differences in cellular interactions, such as the exchange of goods between symbiont A and B and their respective coral host cells. Experimentally linking these molecular mechanisms to life-history traits will provide novel insights into the mechanisms used to adapt to the environment through symbiotic partnerships.

To understand why certain pairings have a selective advantage from an evolutionary perspective, we need to systematically study how the variation in cell biological traits is linked to variation in fitness of the 2 endosymbiotic partners (Fig 1). Highest fitness is defined as contributing the most gene copies to future generations; this requires a “life-history strategy” that maximizes fitness in a given ecological condition [3]. Owing to resource limits, organisms have to trade-off investment in multiple fitness-enhancing traits and maximize fitness by a strategic allocation of resources towards self-maintenance, growth, and (a)sexual reproduction [3]. Direct quantification of how cell biological traits vary between optimal and suboptimal symbiont–host pairings and covary with life-history strategies is key to understanding natural selection operating on these symbiotic systems. For example, differences in efficacy of symbiont uptake, differences between partners in integration of cellular functions and nutrient exchange, and differences between symbiont–host combinations in their ability to adjust to environmental change, will all determine which symbiont–host pairings are optimal in a given ecological niche.

To uncover the links between variation in key cellular mechanisms and life-history traits among hosts, symbionts, and host–symbiont combinations as a basis for understanding natural selection in corals, we need a cross-disciplinary approach, using laboratory and field experiments informed by life-history and evolutionary theory. Controlled laboratory experiments are essential to uncover the cell biology of endosymbiosis (the protein-encoded mechanisms that drive symbiont recognition and selection) and functional responses to distinct symbionts. Such a detailed cell biological dissection requires the use of omics in conjunction with targeted candidate-based approaches, quantitative light microscopy, genetic manipulation, and biochemistry. However, corals are difficult to keep, grow slowly, produce nonsymbiotic larvae only once a year, and have long generation times, making these techniques difficult to apply.

To directly link cell biological differences between optimal and suboptimal symbiont–host pairings with life-history traits, experiments can be carried out using *Exaiptasia diaphana* (Aiptasia), a marine anemone coral symbiosis model. Aiptasia is well-suited to such experiments because it easily pairs with different symbionts, enabling researchers to experimentally relate distinct pairings to life-history strategies and fitness [4]. Aiptasia exhibits both asexual and sexual reproduction [5], it is easy to elicit the production of nonsymbiotic larvae, and larvae settle 2 weeks after fertilization, reaching sexual maturity within months [6]. Furthermore, their capacity to release gametes is dependent on symbiosis [6], implying that symbiosis affects its life-history strategies.

A rich molecular toolkit is already available for Aiptasia; in fact, work on Aiptasia has been fundamental in creating the first molecular framework for symbiont recognition and receptor-mediated uptake, host cell immune suppression during symbiont selection, coordination of nutrient provision by the symbiont, host cell signaling and growth, and symbiont-specificity of host cell transcription and metabolism [1]. Unlike other models, Aiptasia can thus readily reveal how cellular crosstalk differs between optimal symbiont–host pairings and suboptimal ones, and how this translates to differences in life-history strategies and fitness.

In parallel to a model system approach, evolutionary theory can be exploited to predict which life-history strategies evolve in which ecological environments [7]. Life-history theory predicts that resources invested in one life-history trait (e.g., reproduction) cannot be invested in another (e.g., self-maintenance). It can also be used to predict how organisms might resolve trade-offs. For example, corals should invest more in self-maintenance and growth when competition for resources is intense, or when offspring survival rates are low [7,8]. By combining evolutionary theory and cell biology, we can start to predict host and symbiont life-history strategies for key ecological conditions, and how those are related to underlying molecular principles. Such predictions will inform optimal experimental designs required for understanding coral ecology and evolution. The proposed predictive framework has general applicability: it generates predictions at the level of taxa, species, populations, and individuals alike [9]. Because life-history theory predicts different strategies under different ecological conditions [3,7,8], we would expect host–symbiont pairings to vary across those key biological levels, e.g., species or populations (as is evident from empirical data [10]). For example, the 3D structure of marine habitats creates macro- and micro-environmental variations in factors such as current strength (thus nutrient availability) and light intensity (thus photosynthesis). Any such factors affecting mean and/or variance of offspring versus adult survival will affect optimal life-history strategies [3].

This rich vein of research questions is best approached using experimental “evolution lines” [11] designed to show how key ecological factors affect the microevolution of cell biological mechanisms associated with endosymbiosis. Each replicated evolution line could have a set mean/variance in a key (a)biotic factor (e.g., nutrient availability, light level, strength of intraspecific competition) to which an experimental population of hosts and symbionts is exposed over multiple generations. Such experiments will reveal causal links between ecology, life-history, partner reward, and the cellular mechanisms underlying optimal host–symbiont pairs, and thus reveal how ecology shapes the diversity of host–symbiont interactions observed in nature. Such laboratory-based research should be combined with observational field studies asking whether the types of host and symbionts predicted to (co-)evolve in a given ecological condition indeed occupy the expected ecological niches in the wild and field experiments using mesocosms. Such fieldwork is key, because only by comparing field-based ecological and lab-based insights will we be able to understand the ecological and cell biological diversity present in nature.

Coral reefs are greatly threatened by climate change. In particular, coral bleaching due to elevated sea water temperature leads to coral death with devastating consequences for ecosystem function and biodiversity. The development of a predictive framework for the evolution of coral symbiosis will firmly connect cell biology with ecological and evolutionary theory to better understand the resilience and capacity of corals to adapt to a changing environment.
